# Tissue Specific Transcriptome Changes Upon Influenza A Virus Replication in the Duck

**DOI:** 10.3389/fimmu.2021.786205

**Published:** 2021-11-05

**Authors:** Lee K. Campbell, Ximena Fleming-Canepa, Robert G. Webster, Katharine E. Magor

**Affiliations:** ^1^Department of Biological Sciences, University of Alberta, Edmonton, AB, Canada; ^2^Li Ka Shing Institute of Virology, University of Alberta, Edmonton, AB, Canada; ^3^Division of Virology, St. Jude Children’s Research Hospital, Memphis, TN, United States

**Keywords:** RNA-seq, highly pathogenic avian influenza, proinflammatory cytokines, reservoir host, duck (*Anas platrhynchos*)

## Abstract

Ducks are the natural host and reservoir of influenza A virus (IAV), and as such are permissive to viral replication while being unharmed by most strains. It is not known which mechanisms of viral control are globally regulated during infection, and which are specific to tissues during infection. Here we compare transcript expression from tissues from Pekin ducks infected with a recombinant H5N1 strain A/Vietnam 1203/04 (VN1203) or an H5N2 strain A/British Columbia 500/05 using RNA-sequencing analysis and aligning reads to the NCBI assembly ZJU1.0 of the domestic duck (*Anas platyrhynchos*) genome. Highly pathogenic VN1203 replicated in lungs and showed systemic dissemination, while BC500, like most low pathogenic strains, replicated in the intestines. VN1203 infection induced robust differential expression of genes all three days post infection, while BC500 induced the greatest number of differentially expressed genes on day 2 post infection. While there were many genes globally upregulated in response to either VN1203 or BC500, tissue specific gene expression differences were observed. Lungs of ducks infected with VN1203 and intestines of birds infected with BC500, tissues important in influenza replication, showed highest upregulation of pattern recognition receptors and interferon stimulated genes early in the response. These tissues also appear to have specific downregulation of inflammatory components, with downregulation of distinct sets of proinflammatory cytokines in lung, and downregulation of key components of leukocyte recruitment and complement pathways in intestine. Our results suggest that global and tissue specific regulation patterns help the duck control viral replication as well as limit some inflammatory responses in tissues involved in replication to avoid damage.

## Introduction

Influenza A virus (IAV) causes disease in both humans and animals, resulting in periodic epidemics and potentially global pandemics. Mallard ducks (*Anas playrhynchos*) are the natural host and reservoir IAV, and as such are highly resistant to viral pathology or mortality while still being permissive to viral replication (1–3). In birds, the virus is categorized as either highly or low-pathogenic avian influenza (HPAI or LPAI, respectively) depending on the outcome of infection in chicken (4, 5). Ducks are resistant to both HPAI and LPAI viral strains of IAV, although it is of note that some H5 strains can cause severe pathology or even mass die offs in ducks (6–8). HPAI strains replicate in the lungs of infected ducks and chickens causing more pathology to infected animals, and these strains can also cause systemic dissemination of viral particles (7, 9). LPAI strains replicate in the intestines of ducks to high titers without causing serious lesions (1, 10). This adaption allows the virus to be spread in excrement, and transferred through shared waterways, or when ducks fly over poultry farms, giving the ducks the moniker of the “Trojan horses” of infection (11). In particular, H5Ny strains of influenza continue to be enzootic in ducks and remain of concern for their pandemic potential (12).

The duck mounts a robust immune response to IAV, involving key viral detectors and effectors, as recently reviewed (13–15). Key to the duck’s innate defense is the cytoplasmic sensor DExD/H-Box Helicase 58/retinoic acid-inducible gene I (*DDX58*/RIG-I) which detects IAV, and the mitochondrial antiviral-signaling protein (MAVS) signaling pathway. Notably, components of this pathway differ between ducks and chickens (5, 16–22). We have postulated that RIG-I being absent in chickens is one of the main reasons why chickens are so susceptible to IAV when compared to ducks (17).

While there are significant differences in basal expression of duck innate receptors and downstream effectors between tissues (15), it is still unknown how different tissues contribute to resistance to IAV and yet control damage from IAV infection despite high viral load. As ducks share a long evolutionary history with IAV (2) it is likely that global changes, not just the immune response, contribute to protection. Additionally, due to constant selective pressures from the virus, the duck may have unique antiviral effectors or splice isoforms. By comparing immune responses in tissues during replication we may discern differences in global response to avian influenza in ducks that are key to surviving highly pathogenic viruses that replicate systemically, while also permitting replication of harmless strains in intestine.

Several groups have examined gene expression in duck tissues following challenge with H5N1 strains of influenza. Transcriptome sequencing was performed on Shaoxin ducks infected with high and low pathogenic H5N1 strains (23), but this study was limited to lung tissues. Kumar and colleagues examined genome wide gene expression patterns to high and low pathogenic H5N1 viruses in ducks, and compared lung transcriptomes at 5 days post-infection (24). Smith and colleagues sequenced RNA from domestic Gray mallards and chickens infected with high and low pathogenic H5 strains (25) and focus on gene expression contributing to species differences in IAV susceptibility. However, these studies did not compare differences in gene expression between duck tissues involved in viral infection.

In our previous study comparing duck responses to highly and low pathogenic viruses, ducks infected with rgA/Vietnam 1203/2004 (H5N1) and A/British Columbia 500/2005 (H5N2) upregulated key innate immune genes, and although we characterized only a limited number of genes using qPCR, the ducks rapidly cleared both viruses with robust early responses (26). Our aim is to extend this study by aligning pair-ended RNA-seq data to the current Pekin duck genome assembly (ZJU1.0) to analyze the global differential expression patterns in tissues involved in viral replication (lungs or intestine) and the lymphatic response (spleen) and identify novel candidate genes for future exploration.

Here we obtain transcriptome information from polyadenylated RNA of ducks infected with a reverse genetics version of the highly pathogenic H5N1 strain rgA/Vietnam/1203/04 (VN1203) and the low pathogenic H5N2 strain A/mallard/BC/500/05 (BC500). We look at the global differential expression (DE) in lung and spleen of Pekin ducks infected with VN1203; and lung, spleen and intestines of ducks infected with BC500. We have highlighted differences and similarities in differential expression of transcripts and identified sets of genes that have arisen by duplication and may contribute to host specific resistance. Our results suggest that tissue specific regulation mechanisms may play an integral role in both providing protection against IAV replication while limiting inflammatory responses.

## Methods

### Viral Infection and RNA Collection

Viral infection and tissue collection were described previously (17, 27). Briefly, the VN1203 strain A/Vietnam/1203/04 (H5N1) was recreated using reverse genetics (28), while the BC500 strain A/mallard/BC/500/05 (H5N2) was collected during routine surveillance of wild ducks in British Columbia, Canada. Outbred 6 week old Pekin ducks from Metzer Farm or Ideal Poultry were used for this study. Ducks were mock treated with PBS or infected with 10^6^ of 50% egg infectious doses (EID_50_) of VN1203 or BC500 using the natural route of infection by dripping virus in nares, eyes and trachea. Viral replication was tracked by taking tracheal and cloacal swabs from some ducks, and reported previously (27). For BC500, cloacal swabs were positive at 2 and 3 days post-infection (dpi), and tracheal swabs were negative. For VN1203, tracheal swabs were positive at 3 dpi, while cloacal swabs were negative. Influenza RNA quantification showed M gene was highly expressed in lung and spleen for all three days in VN1203 infected ducks (26). At 1, 2 or 3 dpi, ducks were euthanized, tissues were harvested and RNA was extracted using TRIzol reagent (Invitrogen). Samples were DNAse treated and stored at -80°C. Lung, spleen and intestine samples from mock treated animals were collected from 3 ducks at each time point (1, 2 and 3 dpi), however due to RNA quality, only 1 duck from mock infected animals on 2 dpi was used. RNA samples from lung and spleen from VN1203 infected ducks were used from 4 ducks at each time point, while RNA samples from lung, spleen and intestines from BC500 infected ducks were from 3 ducks at each time point. The sex of each duck was determined from raw read counts of the *SWIM6* gene (LOC101797738) in each RNA sample. *SWIM6* is located on the W chromosome found in WZ females, but not ZZ males, and was established as a valid determinant of avian embryo sex by He and colleagues (29). A table of each sample name and corresponding sex of the duck can be found in **Supplementary File 1**.

### Library Construction and Sequencing

Library preparation and poly-adenylated RNA sequencing were performed by LC Sciences (https://www.lcsciences.com/). Briefly, RNA-seq paired end libraries were created using Illumina’s TruSeq-stranded-mRNA sample preparation protocol (Illumina, San Diego, CA). Integrity of RNA was checked using an Agilent Technologies 2100 Bioanalyzer. Two rounds of purification of poly(A) containing mRNAs were performed using oligo-dT magnetic beads. cDNA libraries were made and quality was assessed using Agilent Technologies 2100 Bioanalyzer High Sensitivity DNA Chip. Paired-ended sequencing of the cDNA libraries was performed using llumina’s NovaSeq 6000 sequencing system. The sequencing resulted in paired 150 bp reads with approximately 6GB of data per run, resulting in a sequencing depth of approximately 40 million reads per sample.

Sequence data was submitted to NCBI sequence read archive (SRA) under the BioProject ID PRJNA767080.

### Sequence Analysis and Differential Expression

LC Sciences used CutAdapt (30) to remove adaptors and low-quality bases and then verified for quality using FastQC (Available online at: http://www.bioinformatics.babraham.ac.uk/projects/fastqc/). We used Trimmomatic (31) on these reads to further separate paired end reads and remove unpaired reads from the data. RNA sequence reads were aligned to the NCBI genome of the mallard duck (*Anas platyrhynchos*, assembly ZJU1.0) using HISAT2 version 2.20 (32). Reads were counted and sorted using FeatureCounts *version 2.0.0* (33). Due to the overall rate of unassessed gene duplications in the genome, multi-mapped reads were counted and fractionally assigned to features. Reads were sorted by “feature” for differential expression between exons, and “Meta-feature” for differential expression between transcripts.

Differential expression (DE) analysis was performed in the R studio environment *version 4.0.0* using EdgeR (34). Library sizes were normalized using the trimmed mean of M-values (TMM). Fisher’s exact test was used to determine the number of DE genes of infected tissues compared to their internal control samples. Genes were considered DE if FDR<0.05 and fold change (FC) >2 and were used in downstream analysis. All DE genes for both VN1203 and BC500 experiments can be found in **Supplementary File 2**. Venn diagrams comparing expressed genes were made using DiVenn (35) or Venny 2.1 (36).

The EdgeR function diffspliceDGE was used to assess alternative splicing events during infection. All alternatively spliced genes for both VN1203 and BC500 experiments can be found in **Supplementary File 3**.

### Enrichment and STRING Analysis

Differentially expressed genes were combined for all three days of infection in each different tissue, infected with VN1203 or BC500. These genes were subjected to over-representation analysis (ORA) for gene ontology biological process (GO BP) terms, with the noRedundant filter added to reduce redundant enrichment terms on WebGestalt (37).

Search Tool for the Retrieval of Interacting Genes/Proteins (STRING) diagrams were made using Cytoscape version 3.8.0 (38). Enrichment analysis of differentially expressed genes (DEGs) was performed in Cytoscape using the STRING app for STRING diagrams and enrichment (39). Due to the relative incompleteness of avian enrichment and interaction databases, all gene names were changed to the human equivalent and searched against the human database.

### Gene Annotation and Identification

Many of the annotations in the NCBI file had only numerical descriptions, and not annotated gene names. To assign names to those with significant hits from the DEG lists, we searched on NCBI and if available from the description, the gene name was manually assigned. Gene names were changed using a find and replace macro in excel (**Supplementary File 4**) written by www.extendedoffice.com (https://www.extendoffice.com/documents/excel/1873-excel-find-and-replace-multiple-values-at-once.html). Any genes flagged in the datasets named with location numbers only were submitted to NCBI BLAST to search against both the bird and mammalian databases. For genes without significant BLAST orthologs, the location number identifier was not changed. Genes flagged as significant which had the same gene name as others in the list were investigated to determine if this was a genome mis-assembly, paralogous gene or misidentification through BLAST, using protein alignments, and chromosomal locations.

To differentiate genes in our results we applied certain rules to naming related genes. Genes with the “pseudogene” designation in NCBI were assigned a “-pseu” suffix. Likewise, genes classified as noncoding RNA (ncRNA) were given a “-ncRNA” suffix. Genes which were identified as “like” another gene on NCBI were given the “-L” suffix. In cases where multiple genes in one dataset were classified with the same name with the “-L” suffix, the genes were numbered as “-L#”, with the lowest number being closest in chromosomal location to the presumed ortholog or the annotated gene on NCBI. Genes which shared identical names, that could not be clarified by the above methods kept their gene names but were given a numerical suffix in the order of the genes on their respective chromosomes (i.e. “*PARP14.1*, *PARP14.2*).

## Results

### RNA Sequencing Reads From Ducks Infected With VN1203 or BC500 Align to Genome and Cluster by Tissue and Virus Infection

**Figure 1:**
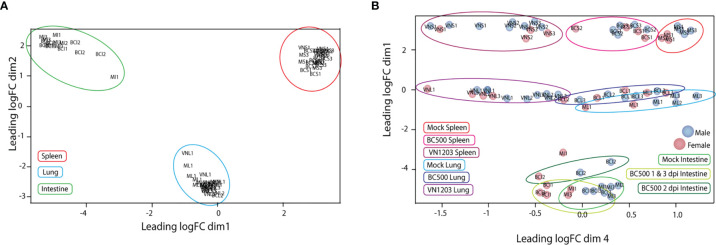
Multidimensional scaling (MDS) plot of normalized individual RNA-sequencing experiments. MDS plots were made in the EdgeR program comparing the top 200 logFC results between each sample. Individual samples and their relative similarity and differences were compared using dimensions 1 and 2 **(A)** and dimensions 1 and 4 **(B)**. Individual plot points were named by treatment (VN, VN1203; BC, BC500 and M, Mock), tissue (L, Lung; S, Spleen and I, intestine) and dpi (1, 2 and 3). Ex: Spleen from mock treated duck 1 dpi = MS1. Individual male ducks are identified by a blue dot and females by a red dot.

To determine the quality of our RNA reads and amount of coverage over the duck genome, we aligned our RNA-seq data to the NCBI *Anas platyrhynchos* genome assembly ZJU1.0. The average alignment rate of RNA sequencing reads to genome was 92%. Counted and sorted reads were successfully assigned to features at an average rate of 75%, indicating good coverage and sequencing depth of samples. The counted and assigned reads for each sample when graphed using multidimensional scaling (MDS) clustered with similar tissue (**Figure 1A**) and virus type (**Figure 1B**). Samples from lung and spleen tissues from VN1203 infected ducks were very distinct from tissues from BC500 infected and mock treated ducks, while tissues from infected ducks were distinct from mock treated samples. As intestine samples were taken only from ducks infected with BC500, we expanded our MDS analysis to include day post infection (dpi), where 1 and 3 dpi clustered more closely than 2 dpi. One mock treated intestine sample (MI1) was an outlier in this group, however as it clustered with other intestine samples, and did not significantly change the overall analysis when removed (data not shown), we kept it in in the data set for further analysis. Sex of ducks is indicated by color coding of samples (**Figure 1B**). Of note, control birds were a mix of male versus female ducks (4:3). Ratios of male to female ducks infected with LPAI were 1:2, 2:1, and 2:1 on 1, 2 and 3 dpi respectively. Ratios of male to female ducks infected with HPAI were 3:1, 3:1 and 2:2 on 1, 2 and 3 dpi. The MDS plot suggests some segregation of samples according to sex.

### VN1203 Infected Ducks Have More Differential Gene Expression Than Those Infected With BC500

To evaluate the number of genes differentially expressed in lungs, spleens and intestines of ducks infected with VN1203 or BC500, we used Fisher’s exact test to compare infected tissues to control on 1, 2 and 3 dpi. Lung and spleen samples from VN1203 infected ducks had the most statistically significant (FDR<0.05) DEGs on all three days of infection, while BC500 infected ducks had much less DE in lungs and spleen (**Table 1**). We have previously reported much higher transcript levels following VN1203 than BC500 infection when analyzing expression of individual immune genes (26). In contrast to other tissues sampled in BC500 challenged ducks, the intestines had more DEGs, although most were below the threshold for fold change cutoff (**Table 1**).

**Table 1:** Differentially expressed genes in tissues from ducks infected with VN1203 or BC500.

	Total DEGs (FDR < 0.05)	Total DEGs (FC >2 and FC <-2)	Upregulated (FC >2)	Downregulated (FC <-2)
**VN1203 - Lung 1 dpi**	4204	1804	1063	741
**VN1203 - Lung 2 dpi**	2808	1235	542	693
**VN1203 - Lung 3 dpi**	4719	1939	772	1167
**VN1203 - Spleen 1 dpi**	4894	2738	1054	1684
**VN1203 - Spleen 2 dpi**	3572	1575	698	877
**VN1203 - Spleen 3 dpi**	4465	2121	943	1178
**BC500 - Lung 1 dpi**	53	51	50	1
**BC500 - Lung 2 dpi**	110	93	82	11
**BC500 - Lung 3 dpi**	10	10	10	0
**BC500 - Spleen 1 dpi**	84	75	72	3
**BC500 - Spleen 2 dpi**	320	217	202	15
**BC500 - Spleen 3 dpi**	75	65	55	10
**BC500 - Intestine 1 dpi**	73	64	55	9
**BC500 - Intestine 2 dpi**	3732	1583	551	1032
**BC500 - Intestine 3 dpi**	401	187	106	81

Total DEGs were sorted by false discovery rate (FDR) <0.05. Genes were considered upregulated if the log2(FC)>1 and downregulated if the log2(FC)<-1.

**Figure 2:**
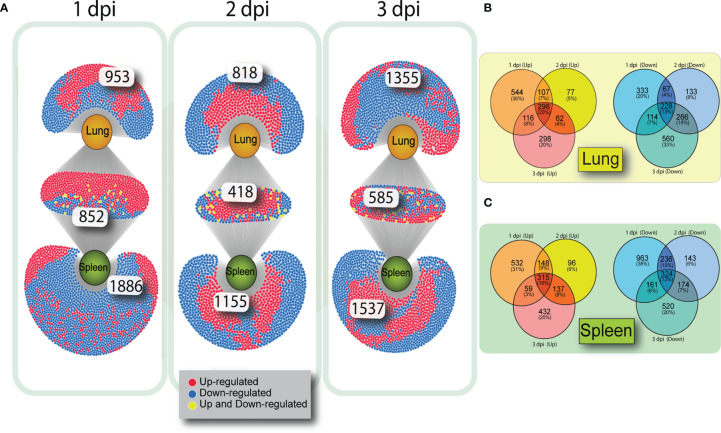
Venn diagrams showing overlap of gene expression on 1, 2 and 3 dpi in VN1203 infected ducks. Differentially expressed genes were assigned up or down regulated based on log2(FC) (upregulated>1, downregulated<-1) and overlap of DEGs was compared between tissues using DiVenn **(A)**. Overlap of up or downregulated gene populations specific to each tissue in ducks infected with VN1203 in lung **(B)** or spleen **(C)** created using Venny 2.1.

To determine how many genes were similarly upregulated or downregulated on all three days of infection, genes were filtered by FDR (>0.05) and log2(FC). Although each tissue had a number of unique altered genes there was considerable overlap of DE genes in lung and spleen on each day post infection (**Figure 2A**). At 1 dpi with VN1203, 852 DE genes are common between lung and spleen, with most genes upregulated. However, at 3 dpi the number of DEGs unique to lung are greatly increased.

To determine if the same subsets of genes were either up or down regulated on all days of VN1203 infection, we created VENN diagrams to compare gene regulation within each tissue. Genes that were up or downregulated during VN1203 infection in lung (**Figure 2B**) or spleen (**Figure 2C**) were analyzed to see how many were expressed during all 3 dpi, and how many changed expression depending on day. In the lung only 20% of upregulated genes were upregulated on all days of infection. This is similar in the spleen, where 18% of genes were upregulated on all days of infection. In both lung and spleen, the most DE specific to day of infection happens on 1 dpi (36% in lung, 31% in spleen), while 2 dpi has the lowest number of genes specific to that day differentially expressed (5% in lung, 6% in spleen). A similar pattern emerges for the downregulated genes, as only 13% of the downregulated DEGs were downregulated all days of infection in both lung and spleen. Once again, 2 dpi had the lowest number of downregulated DEG specific to that day (8% in lung, 6% in spleen).

### GO Biological Process Enrichment Analyses Find Commonalities and Dissimilarities Between VN1203 and BC500 Infection

**Figure 3:**
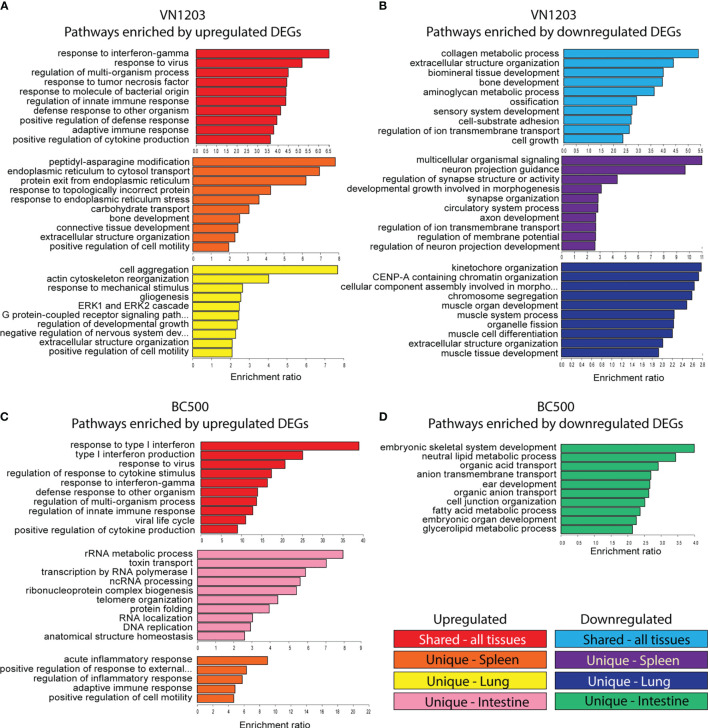
Gene ontology (GO) analysis of common and uniquely differentially expressed transcripts between VN1203 and BC500 infected ducks Differentially expressed genes were assigned up or down regulated based on log2(FC) (upregulated>1, downregulated<-1) lists and were arranged as “similar between tissues”, “unique to spleen”, “unique to lung” or “unique to intestine”. For ducks infected with VN1203, the top 10 most highly enriched terms in the GO biological function category were graphed for upregulated genes **(A)** and downregulated genes **(B)**. For ducks infected with BC500, the top 10 most highly enriched terms derived from upregulated genes **(C)** or downregulated genes **(D)** were identified and enrichment ratio for each term is graphed.

To determine which pathways were enriched in each tissue during infection with VN1203 or BC500, we submitted DE genes to the WebGestalt server. All DE genes were clustered together to allow for analysis of enriched GO terms on all three days post infection, and the top 10 statistically significant (FDR<0.05) hits were reported in **Figure 3**. Results were separated into terms in common (GO terms enriched in all tissues sampled) or unique to tissue (spleen and lung in VN1203 infection, spleen, lung, and intestine for BC500 infection). During VN1203 infection, most of the commonly enriched pathways represented by upregulated DEGs were involved in immune responses (**Figure 3A**). Both innate and adaptive immune terms were enriched (GO:0045088 and GO:0002250). Likewise, response to virus was also highly enriched (GO:0009615). Pathways that were enriched by unique upregulated DEGs in spleen included many terms involved in protein folding, and misfolded protein responses (GO:0034976, GO:0035966, GO:0032527 and GO:0018196). Likely, these pathways and those involved in cell motility (GO:2000147) are enriched due to immune cell accumulation and activation. The lung of VN1203 infected ducks was enriched with terms involving actin and cytoskeleton rearrangement (GO:0031532 and GO:0043062), and cell surface signaling pathways (GO:0007186).

GO term enrichment resulting from downregulated DEGs found common terms in cell adhesion (GO:0031589) and cell growth (GO:0016049) (**Figure 3B**). Interestingly, bone mineralization or ossification terms were also enriched in the common downregulated DEG population (GO:0060348, GO:0001503 and GO:0060348). GO terms enriched in spleens of VN1203 infected ducks due to downregulation were mostly involved in neuronal signaling and cell development, while terms enriched by downregulated DEGs in lungs were comprised of pathways involved in muscle development (GO:0060537, GO:0042692, GO:0007517 and GO:0003012) and cell cycle progression (GO:0007059, GO:0061641 and GO:0051383).

DEGs upregulated during BC500 infection enriched similar pathways as those found in VN1203, however BC500 enriched the type I interferon pathway the most (**Figure 3C**). Surprisingly, upregulated DEG in the intestine enriched many pathways involving not only cell cycle progression, but RNA processing. Due to the overall low number of DEGs in lungs infected with BC500, there were not enough uniquely expressed DEGs to properly analyze enrichment. Upregulated genes in BC500 infected spleens only enriched five pathways and all of those were involved in immunity. There were not enough total down regulated DEGs to analyze common enrichment or unique enrichment for spleens and lungs (**Figure 3D**). The intestines did have unique enrichment of many pathways involved in metabolic processing of glycerolipids and fatty acids (GO:0046486 and GO:0006631) among the downregulated DEGs.

### VN1203 Infection Greatly Increases Alternative Splicing Events in Lung

To determine if IAV infection alters alternative splicing (AS) events in lung, spleen and intestine of infected ducks, we analyzed alternative splicing events by counting differences in reads mapped to individual exons between infected and control samples. As with total DE expression, VN1203 infected lungs and spleens had many more AS events than BC500 infected tissues (**Table 2**), with infected lungs having the most AS events on all 3 days post infection. Lungs from ducks infected with VN1203 had 456, 50, and 267 AS events at 1, 2 and 3 dpi. Spleens from VN1203 infected ducks had 70, 57, and 106 AS events on 1, 2 and 3 dpi respectively. Interestingly, while intestine tissues from BC500 infected ducks had a much greater amount of total DE genes than other tissues infected with the same virus, intestines only had 7, 37 and 9 AS events detected on 1, 2 and 3 dpi (respectively). As IAV proteins, such as NS1, can influence AS events in the cell (40), it is possible that VN1203 infection itself is responsible for this dramatic increase in AS events.

**Table 2:** Counts of alternatively spliced (AS) transcripts in tissues of ducks infected with VN1203 or BC500.

	Total AS Genes (Simmes FDR < 0.05)
**VN1203 - Lung 1 dpi**	456
**VN1203 - Lung 2 dpi**	50
**VN1203 - Lung 3 dpi**	267
**VN1203 - Spleen 1 dpi**	70
**VN1203 - Spleen 2 dpi**	57
**VN1203 - Spleen 3 dpi**	106
**BC500 - Lung 1 dpi**	7
**BC500 - Lung 2 dpi**	14
**BC500 - Lung 3 dpi**	7
**BC500 - Spleen 1 dpi**	14
**BC500 - Spleen 2 dpi**	15
**BC500 - Spleen 3 dpi**	21
**BC500 - Intestine 1 dpi**	7
**BC500 - Intestine 2 dpi**	37
**BC500 - Intestine 3 dpi**	9

Individual exon counts in VN1203 or BC500 infection were compared to controls. Alternative splicing events were considered significant (FDR<0.05) when analyzed using EdgeR diffSpliceDGE program, using the “Simmes” method.

To determine the GO terminology associated with these AS events, all significant (FDR < 0.05) AS genes were submitted to the WebGestalt server. Only VN1203 infected ducks had enough genes to produce statistically significant (FDR < 0.05) GO results (**Supplementary Figure 1**). Lungs of ducks infected with VN1203 at 1 dpi have AS events in genes associated with muscle cell proliferation and migration (GO:0033002 and GO:0014812) (**Supplementary Figure 1A**). Curiously, respiratory system terms are also enriched by these AS genes (GO:0060541 and GO:0030323). Both spleens and lungs of VN1203 ducks had statistically significant enrichment of GO terms on 3 dpi (**Supplementary Figure 1B**). There were distinct subsets of GO terms enriched in each tissue, with lung AS genes enriching more general terms associated with actin organization (GO:0007015), protein signal transduction (GO:0051056 and GO:0007265) and general cellular and tissue growth processes. AS events in the spleen however, enriched terms involving inflammatory responses (GO:0002526 and GO:0050727), platelets (GO:0002576), humoral immune responses (GO:0006959) and migration of leukocytes (GO:0050900).

The lack of statistically significant GO terms on other days in lung and spleen is likely due to the relatively low number of genes associated with AS events as well as the various functions of these genes. Repeating the RNA-sequencing with a greater sequencing depth would likely result in a more accurate sampling of AS events in these tissues.

### VN1203 and BC500 Infections Upregulate Shared Sets Of Genes In Spleens, Lungs, and Intestines

**Figure 4:**
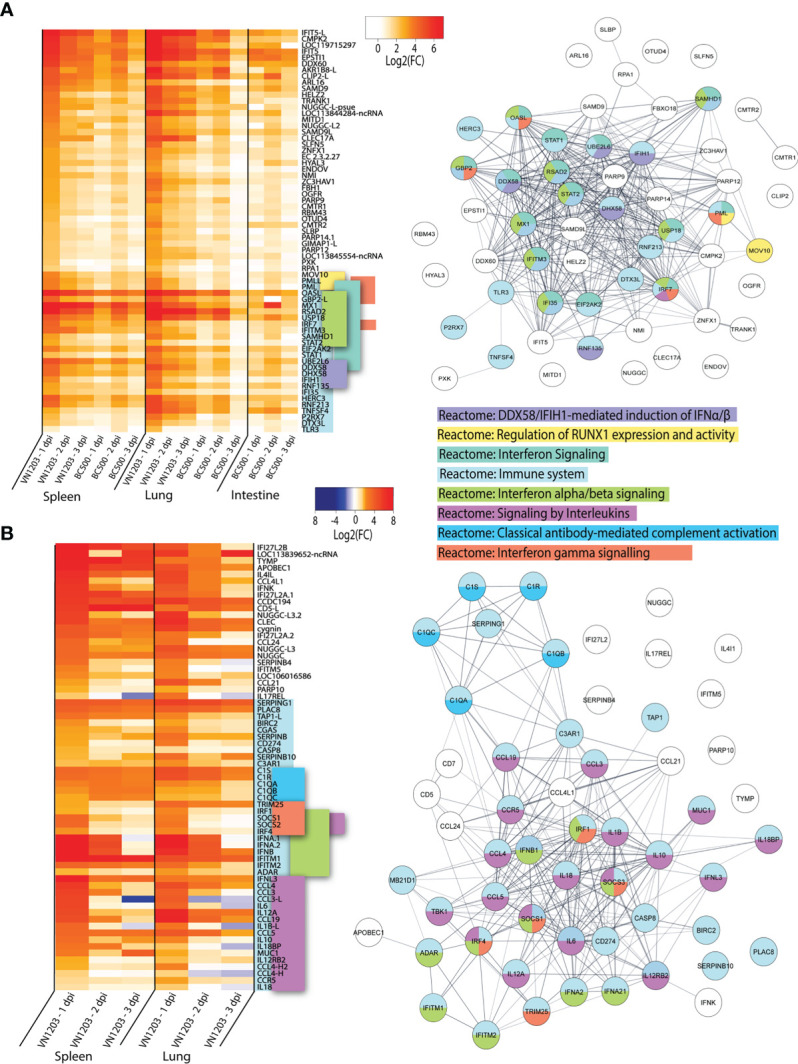
VN1203 and BC500 upregulate genes shared between all tissues sampled. Lists of statistically significant (FDR<0.05) genes were compared for similarity in expression (Log2(FC)>1) and clustered together if they were expressed in **(A)** all tissues in VN1203 and BC500 infected ducks or **(B)** spleens and lungs of VN1203 infected ducks. STRING diagrams depict predicted protein-protein interactions between the products of each gene, while colour coding is based on term enrichment of Reactome pathways. No colour means that the genes did not enrich any of the Reactome terms. Reactome terms were considered significant if FDR<0.05. Enrichment and STRING diagram was created using the STRINGTIE app in Cytoscape.

To determine which genes were shared in response to virus in all three tissues sampled, we inspected the lists of DEGs on 1, 2 and 3 dpi for each tissue infected with either VN1203 or BC500 for genes in common. Between the VN1203 and BC500 infected ducks, there were 65 upregulated shared genes (**Figure 4A**). We subjected this set of genes to Reactome enrichment analysis using Cytoscape STRING app (**Figure 4A**). The largest group of genes enriched the “Reactome: Immune system” pathway. The expression patterns of genes in this group are different between VN1203 and BC500 infection (**Figure 4A**). VN1203 caused highest gene expression at 1 dpi while BC500 infected ducks had highest expression of most of these immune genes at 2 dpi. Indeed, when looking at statistical significance of these genes, the FDR is < 0.05 for most of these genes at 2 dpi in spleen, lung and intestine (**Supplementary File 1**), however this is not the case for most genes 1 and 3 dpi. The large increase of DEGs in ducks infected with BC500 at 2 dpi corresponds to viral titers, as we previously reported that these ducks had cloacal swabs negative for virus on 1 dpi, with significant viral titers on 2 and 3 dpi (27).

To determine which highly upregulated genes were in common between different tissues in VN1203 infected ducks, we filtered significant DEGs by log2(FC)>1.5. Genes that reached these parameters on 1, 2 or 3 dpi were kept, combined into a list for each tissue, then compared. We removed the 65 genes found upregulated in all tissues during both VN1203 and BC500 infection to reduce redundancy in the datasets. Repeat non-coding RNA (ncRNA) and pseudogenes were also removed. A heatmap of most upregulated DEGs in VN1203 infection was plotted showing genes corresponding to different Reactome pathways placed together (**Figure 4B**). The resulting STRING diagram demonstrates that most of the protein products of these genes have predicted interactions (**Figure 4B**). Out of the 67 total genes shared between tissues in VN1203 infection, 44 were associated with the “Reactome: Immune system” pathway. The genes enriched in the classical complement pathway have slightly different expression patterns between lung and spleen. In lungs many of these genes were upregulated 1 dpi, whereas in spleen the expression of these complement genes was sustained through all 3 days. Genes which enriched the “Reactome: Signaling by interleukins” pathway were predominantly upregulated 1 dpi. Proinflammatory interleukins IL-6, IL-1β and IL-18 are all upregulated 1 dpi, consistent with our previous qPCR analyses (41). Genes that enriched both the IFNα/β and IFNγ signaling pathways were mostly upregulated in both tissues 1 dpi. Some, such as *IFNB* and *IFITM1* show sustained expression on all 3 days pi.

Ducks infected with both VN1203 and BC500 upregulate 65 genes in common, most of which produce proteins that respond to interferons or have immune function. VN1203 strongly upregulates a different subset of genes, including genes involved in the complement cascade, proinflammatory cytokines and interleukins and various other genes involved in immune responses. While VN1203 upregulates most immune genes on 1 dpi, BC500 upregulates these genes on 2 dpi, the delay likely due to the time needed for virus to reach the intestine for replication.

Unfortunately, there were no available enrichment databases that accurately placed all genes in enrichment categories. Many genes that are involved in immunity, antiviral defense or are interferon inducible are not yet added to the Reactome databases or without enough additional terms to be considered enriched in this dataset. Genes such as IFIT5 (42–45), DDX60 (46, 47), SAMD9 (48) and SAMD9L (49) are potentially involved in innate immunity or antiviral defense, yet are unclassified in this dataset.

### VN1203 Infection Preferentially Upregulates PRR and Signal Transduction Genes in the Lung and Interleukins in the Spleen of Infected Ducks

To determine which highly upregulated genes were uniquely expressed in lung or spleen of ducks infected with VN1203, we filtered significant DEGs by log2(FC)>1.5 for all three days post infection. Genes which were found in both tissues were removed from the dataset. We removed the 65 genes found upregulated in all tissues during both VN1203 and BC500 infection, as well as repeat ncRNA and pseudogenes, as described previously.

**Figure 5:**
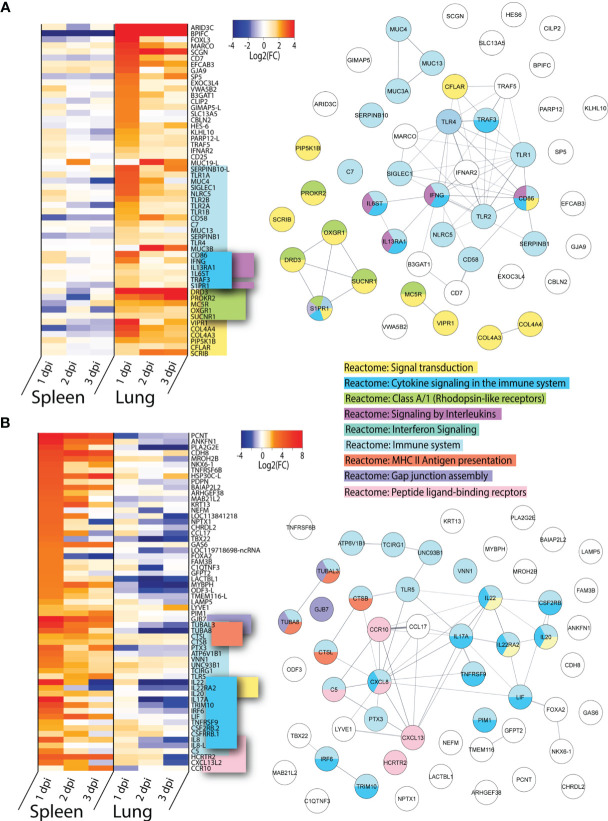
Genes uniquely upregulated in lungs or spleens of VN1203 infected ducks. Lists of statistically significant (FDR<0.05) genes were compared for similarity in expression (Log2(FC)>1.5) and clustered together if they were uniquely upregulated in **(A)** lungs of VN1203 infected or **(B)** spleens of VN1203 infected ducks. STRING diagrams depict predicted protein-protein interactions between the products of each gene, while colour coding is based on term enrichment of Reactome pathways. No colour means that the genes did not enrich any of the Reactome terms. Reactome terms were considered significant if FDR<0.05. Enrichment and STRING diagram was created using the STRINGTIE app in Cytoscape.

Many of the genes upregulated in lung have immune functions, however, only 20 out the total 54 “unique to lung” genes were found enriched in the “Reactome: Immune system” dataset (**Figure 5A**). Within this cluster there is significant overlap between accessory genes in both the “Reactome: Cytokine signaling in the immune system” and “Reactome: Signaling by interleukins” category. These genes are all upregulated on 1 dpi, with the notable exception of *IFNG*. *IFNG* is primarily upregulated in lungs and has increased expression on both 1 and 3 dpi, but curiously not on 2 dpi. Lungs of ducks infected with VN1203 also see unique expression of many PPRs, including *TLR1A*, *1B*, *2A*, *2B* and *4*. All of these TLRs have the highest expression on 1 dpi. The other primary enrichment pathway in lungs is the “Reactome: Signal transduction” which includes “Reactome: Class A/1 (Rhodopsin-like receptors)”. The expression of these pathways is more varied, with some having highest expression at 1 dpi while others at 3 dpi.

Among the genes uniquely expressed in the spleen of VN1203 infected ducks, almost half enrich the “Reactome: Immune system” pathway (**Figure 5B**). As the spleen is secondary lymphatic tissue, it is perhaps not surprising that genes that enriched the “Reactome: MHC II antigen presentation” pathway were preferentially upregulated here. A subset of these genes also enriched the “Reactome: Cytokine signaling in the immune system” pathway, however unlike in the lung, most of these genes code for interleukins and not accessory proteins. A good proportion of the interleukin genes flagged here enriched the “Reactome Interleukin-20 family signaling” pathway. These genes have peak upregulation on 1 dpi, and interestingly, many are also greatly downregulated in lungs.

VN1203 infection causes upregulation of different and specific subsets of immune genes in the lung and spleen. In lung, more genes involved in pathogen recognition and signal transduction were upregulated, while in spleen gene upregulation is centred around pro-inflammatory interleukins and peptide processing.

### BC500 Infection Causes Up or Downregulation of Distinct Subsets of Genes in the Intestines

**Figure 6:**
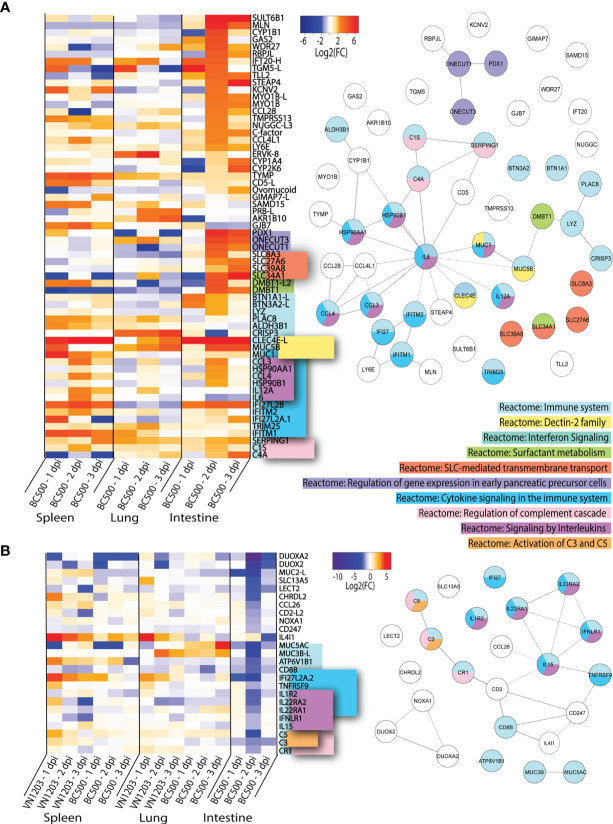
Genes up or downregulated following BC500 infection of ducks. Lists of statistically significant (FDR<0.05) genes were compared for similarity in expression (Log2(FC)>1.5) and clustered together if they were uniquely upregulated in at least 2 out of 3 tissues in BC500 infected ducks **(A)**. Lists of statistically significant (FDR<0.05) downregulated (Log2(FC)<-1) in intestine of BC500 ducks were compared to other tissues of both VN1203 and BC500 infected ducks to determine uniquely downregulated genes in intestine **(B)**. STRING diagrams depict predicted protein-protein interactions between the products of each gene, while colour coding is based on term enrichment of Reactome pathways. No colour means that the genes did not enrich any of the Reactome terms. Reactome terms were considered significant if FDR<0.05. Enrichment and STRING diagram was created using the STRINGTIE app in Cytoscape.

To determine which highly upregulated genes were uniquely expressed in spleen, lung or intestine of BC500 infected ducks, we filtered significant DEGs by log2(FC)>1.5 for all three days post infection (**Figure 6**). Genes which were found in 2 out of 3 tissues were included in this dataset (**Figure 6A**). For this analysis, we removed the 65 genes expressed in all tissues identified in **Figure 4A**. Out of the 64 genes highlighted in this dataset, 22 enriched the “Reactome: Immune system” pathway. While most genes found in this dataset in lung or spleens are also upregulated in VN1203 infection, CRISP3 is upregulated by BC500 in the lung on all 3 dpi, but not at all in VN1203 infection. Many of these genes enrich the term “Reactome: Cytokine signaling in the immune system”. These are mostly specific to spleen and intestines of BC500 infected ducks and peak at 2 dpi. This is also true for a smaller number of genes which enriched the “Reactome: Signaling by interleukins” pathway. A subset of genes enriches the “Reactome: Regulation of genes in early pancreatic cells” pathway. These genes are all transcription factors and are primarily upregulated in intestines at 2 dpi. A group of solute carrier (SLC) family member genes enriched the “Reactome: SLC-mediated membrane transport” term uniquely in intestines from BC500 ducks. These SLC genes are upregulated starting at 2 dpi. Components of the complement pathway enriched the “Reactome: Regulation of complement cascade”, similar to what is seen in VN1203 infection (**Figure 5B**). All three of these genes are upregulated 2 dpi in intestines, while their expression is variable in spleen and lung. It is of note that many of the highly upregulated genes specific to intestine during BC500 infection are not characterized in infection. As with the previous datasets, some genes in this dataset such as *CCL28* (50) are involved in immune cell responses and others such as *LY6E* (51), are interferon stimulated genes (ISGs).

As IAV can replicate in the intestines to high titres without causing significant damage or pathology, we investigated the differences in downregulated genes in the intestines compared to all other tissues to see if downregulation of specific genes might minimize pathology. DEGs in the intestines were filtered by significance (FDR<0.05) and log2(FC) (<-1). From this list, we manually compared genes of interest to infection in lung and spleen of VN1203 and BC500 infected ducks. Genes which were highly downregulated in intestines that may play a role in either immune responses or viral restriction were examined in each tissue and visualized using a heatmap (**Figure 6B**). Many of the genes in this dataset of downregulated genes enrich the Reactome pathway “Reactome: Immune System”. These genes also enrich the “Reactome: Cytokine signaling in the immune system” and “Reactome: Signaling by interleukins”. The downregulation of these genes predominantly happens on 2 dpi, the time point when many other immune genes are upregulated in the intestines of BC500 infected ducks. The Reactome pathway “Reactome: Regulation of complement cascade” is also enriched by a subset of these downregulated genes, however unlike in **Figure 6A**, the genes that enrich this term also enrich the “Reactome: Activation of C3 and C5” pathway. While BC500 infection in the duck causes less DE than VN1203, there are many genes unique to the intestinal response to this virus. Many of these genes are uncharacterized in viral infection and warrant further study.

### Interferon and Cytokine Responses Peak at 1 dpi With VN1203, and ISGs Peak at 2 dpi With BC500

**Figure 7:**
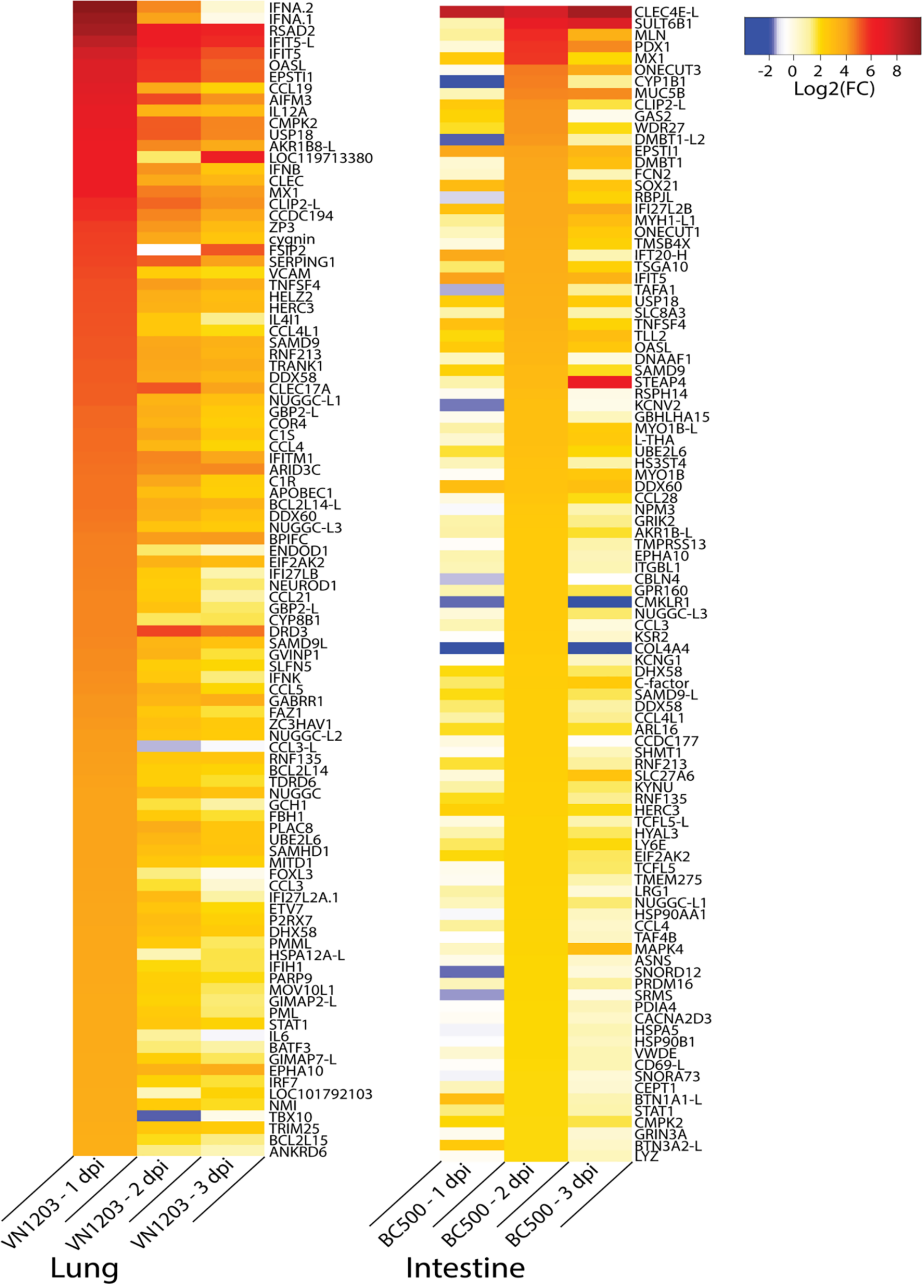
Top 100 upregulated genes in lungs of VN1203 and intestines of BC500 infected ducks. Lists of statistically significant (FDR<0.05) genes were filtered for the top 100 genes expressed in lungs of VN1203 infected ducks on 1 dpi, and intestines of BC500 infected ducks on 2 dpi. Redundant genes, pseudo genes and genes denoted as ncRNA in NCBI were manually removed from lists.

To identify the genes contributing to the peak immune response in the sites of replication, we examined gene expression patterns in infected ducks. Because many important immune genes were upregulated in the lungs of ducks infected with VN1203 on 1 dpi, and most gene regulation in intestines of ducks infected with BC500 was at 2 dpi, we filtered all statistically significant (FDR < 0.05) genes on these days by expression levels and compiled lists of the top 100 most up or downregulated genes. We removed genes designated on NCBI as ncRNA or pseudogenes to limit the lists to genes that presumably code for protein. VN1203 induces a much more robust response than BC500 (**Figure 7**), with half of the top upregulated genes being upregulated by as much as log2(FC) of 4.5 or more, while only the top 13 genes in the BC500 dataset are above a log2(FC) of 4. Many of the top upregulated genes of the VN1203 infected lungs are cytokines (For example: *IFNA*, *IFNB* and *IL12A*) or interferon inducible genes (for example *IFIT5*, *OASL* and *Mx*). The top upregulated genes in BC500 however, are lacking high cytokine gene expression, but still have high expression of some interferon inducible genes (notably *Mx*).

We also expanded this analysis to the top 100 genes most downregulated on 1 dpi in VN1203 infected ducks, and on 2 dpi in BC500 infected ducks (**Supplementary Figure 2**). While initially it appears that downregulation is less robust in the lungs of VN1203 infected ducks, this is only on 1 dpi. Inspection of the data reveals that more significant downregulation of genes in the lungs of ducks infected with VN1203 happens at 2 and 3 dpi.

## Discussion

Here we compare global changes in gene expression of ducks infected with the IAV strains VN1203 or BC500 using RNA-seq analysis. We compared RNA profiles of tissues sampled to identify genes which were similarly upregulated in all infected tissues as well as genes which were uniquely upregulated in specific sites. This enabled us to delineate specific responses in sites of virus replication (lungs for VN1203, intestines for BC500) compared to lymphoid tissues (spleen). Ducks respond to VN1203 infection with a high interferon signature at 1 dpi in lung, yet soon down regulate key proinflammatory cytokines. BC500 infection stimulates the highest gene upregulation at 2 dpi in intestine together with downregulation of leukocyte recruitment cytokines. A global picture emerges of a robust and rapid interferon response to VN1203 in lung, and a significant response to BC500 in intestine, yet both responses were tempered to limit damage. As part of the sequencing effort for the *Anas platyrhynchos* genome, Huang and consortium performed transcriptomic analysis of Shaoxin lung tissues following infection with H5N1 strains (23). They saw a similarly robust interferon response early in infection and elaborated on cytokine and defensin expression. Our work builds on this by investigating the global regulation of genes in lung, spleen and intestine using the most current version of the Pekin duck genome (November 15 2020). Smith and colleagues sequenced lung and intestine RNA from domestic Gray mallards infected with similar viruses, but used single-stranded reads at a lower sequencing depth on 1 and 3 dpi only (25). The Gray mallard ducks also had a much more robust response to VN1203 infection than they did to BC500 infection on 1 and 3 dpi. Many of the highly expressed genes are in common with our results, however the additional depth of sequencing reveals less abundant genes and allows us to analyze alternate splicing in the sampled tissues.

Overall, we found that there were 65 upregulated genes common to all tissues following infection with both viruses, while lungs, spleens and intestines had many genes uniquely differentially expressed. Of these 65 similarly upregulated genes, pattern recognition receptors (PRRs) which can detect RNA viruses were highly upregulated in all tissues. This includes RIG-I and the related interferon induced with helicase C domain 1/melanoma differentiation-associated protein 5 (*IFIH1*/MDA5) and toll-like receptor 3 (TLR3). VN1203 infection induces a much more rapid and robust response of IFN inducible genes (such as *RSAD2*/Viperin and *IFIT5*) in the duck than BC500 infection does. The interferon response peaks at 1 dpi for VN1203, while the response to BC500 peaks at 2 dpi. The expression of genes in the RIG-I pathway from the RNAseq data mostly match their expression profiles we previously determined using qPCR (20, 26, 41), with highest expression of *DDX58*/RIG-I, *IFIH1/*MDA5*, RNF135*/RIPLET*, OASL, IFITM3* and *IRF7* on 1 dpi in ducks infected with VN1203. Exceptions include *IFNB* expression and tripartite motif protein 25 (TRIM25) expression in VN1203 infected ducks. In our previous studies, the qPCR results suggested these two genes were robustly expressed on 1 dpi, and rapidly dropped to basal levels by 3 dpi, while our RNA-seq analysis suggests these genes exhibit sustained expression across all 3 dpi. Likely these discrepancies are due to the different techniques of normalization used between the two studies, with the qPCR experiments normalized to a single gene (GAPDH) and the RNA-seq data normalized using TMM and library size. Additionally, RNA-sequencing data will still count splice variants in its read counts, while qPCR may miss some of these variants due to primer design and placement. Some of the changes in gene expression seen in tissues are likely due to infiltrating immune cells responding to infection. We previously reported aggregates of leukocytes in lung tissues and leukocyte depletion of spleen tissues in VN1203 infected ducks (26). Others have also noted infiltration of immune cells to lung, spleen and intestine following influenza infection (52, 53). We previously showed upregulation of CCL19 and CCL21 chemokines, responsible for homing of dendritic cells and naïve lymphocytes (54), in VN1203 infected lung (55). In this study, we see upregulation of these transcripts on all days following VN1203 infection in both lung and spleen, but only in spleens of BC500 infected ducks. Indeed, CCL19 is one of the most upregulated genes. Higher expression of CCL19 and other ISGs was seen in genotyped Ri chicken lines that were more resistant to H5N1 infection (56).

Infection with BC500 induces the most DEGs on 2 dpi. This is especially evident in the site of BC500 replication, the intestine. This is not surprising, given that cloacal swabs from these ducks were negative for virus on 1 dpi, but they were shedding high titres of virus on days 2 and 3 (27). It seems that the virus replicates in intestine at 2 dpi, and the host tissue responds accordingly. Tracheal swabs from BC500 ducks were also negative and this is consistent with our result of relatively low changes in DEGs in the lung. While this virus produces no observable symptoms in the duck, it replicates to high titres and as such can disseminate into the environment. Since we do not see abundant viral transcripts in the intestinal tissues, either the tissue collection missed the major sites of replication, or the cells actively producing virus are present only transiently or rapidly destroyed. The 2 dpi timepoint was not examined by Smith and colleagues in their RNA sequencing data, however comparable to our data, they found similar genes upregulated at 1 dpi in the ileum of BC500 infected mallards, including *IFIT5*, *ESPTI1*, *MX*, *OASL*, *DHX58* and *SAMD9L* (25).

We see many PRR and IFN-inducible genes upregulated in tissues infected with either VN1203 or BC500. We had previously looked at expression of both ring finger protein 135 (*RNF135*/Riplet) and TRIM25 (26), which both augment RIG-I signaling during viral infection. Here we see that *RNF135* is upregulated by infection with either BC500 or VN1203 in all tissues, while *TRIM25* is upregulated in both lung and spleen during VN1203 infection. We also see DExD/H-Box Helicase 60 (*DDX60*) upregulated by both VN1203 and BC500 in all tissues sampled. In mammals, DDX60 binds RIG-I and promotes RNA binding and downstream type I IFN production during viral infection (47) but has not been studied in birds. We also see strong upregulation of IFN inducible genes such as radical SAM domain-containing 2 (*RSAD2* or Viperin), 2′5′-oligoadenylate snythetase-like (OASL), interferon induced protein with tetratricopeptide repeats 5 (*IFIT5* and *IFIT5-L*, a likely mistake in genome assembly) and *Mx*. *RSAD2*, *OASL* and *Mx* are all highly upregulated by VN1203 and BC500 in all tissues sampled. Recent experiments establish the antiviral function of these duck homologues. Overexpression of duck IFIT5 reduces viral titre at early timepoints but appears to inhibit innate immunity later (57). Duck OASL activates the OAS/RNaseL pathway (58). Similarly, overexpression of duck Viperin reduces viral replication (59).

Although upregulated, some genes may not be functional. For example, *Mx* is highly upregulated at 2 dpi in intestines of BC500 infected ducks, while many other PRR and IFN-inducible genes were only slightly upregulated in that tissue. The function of duck Mx has long been in question, as two alleles showed no antiviral activity *in vitro* (60). Similarly, two members of the interferon induced transmembrane protein family IFITM1 and IFITM2, small proteins capable of preventing viral hemi-fusion of membranes preventing entry, have highly upregulated transcripts in intestine, but we previously showed that neither restricts influenza viruses *in vitro* (18). IFITM1 is mis-targeted to plasma membrane, rather than the endosomal compartment due to a unique insertion in ducks, not seen in chickens. Only IFITM3 restricts influenza viruses, and its expression is high in lung, spleen and intestine. Influenza viruses may exploit these adaptations to preferentially replicate in duck intestines.

Alternative splicing events are prevalent in lungs of ducks infected with VN1203, and rare in tissues of ducks infected with BC500. IAV modifies AS events through many mechanisms (61, 62). Some alternative transcripts may have specific antiviral activity. For example, a short isoform of human nuclear receptor co-activator 7 (NCOA7) was induced by interferon and able to inhibit IAV entry through endosomal fusion (63). The short isoform of NCOA7 was also identified in VN1203 infected lungs and spleens, and BC500 infected lungs. The presence of this NCOA7 isoform in ducks suggests it may have a conserved function of viral restriction in vertebrates. The high number of AS events in VN1203 infected tissues may be due to viral subversion of host response, as well as IFN induction of AS events. Interestingly, when subjected to GO analysis, many of the AS events in the lungs of VN1203 infected ducks enriched terms involved with physiology, rather than immunological responses. AS events in spleens of ducks infected with VN1203 found more enrichment in terms associated with humoral and inflammatory responses. These differences may be due to the abundance of each transcript type in each tissue, as there may be more inflammatory cells activated in the spleen during an infection. Human lung epithelial cells infected with the A/WSN/1933 strain of H1N1 also demonstrated an increase in AS events (61). Thompson and colleagues demonstrated through siRNA screening that some of the alternatively spliced genes also were actively enhancing viral replication, and thus knocking these genes out reduced viral titer in infected cells. Of note, some of these genes are also present in our AS analysis in duck tissues, including: *RAB11F1P3*, *PAXBP1*, *IP6K2* and *TNRC6A*. These genes only show as AS in lungs of ducks infected with VN1203. As little is known about AS responses to infection in birds, future research should involve both sequencing infected duck tissues at a greater depth to capture more AS events and investigating these alternate transcripts in duck cells to determine which aid or restrict IAV replication.

We found gene duplications unique to ducks particularly interesting, especially if the mammalian homologue has known antiviral activity. The poly-ADP-ribose polymerase (PARP) family of genes is largely understudied but is often associated with DNA repair and transcription. We found members of this family upregulated by both VN1203 and BC500 infection in ducks (*PARP9*, *PARP10*, *PARP12* and *PARP14*). Of interest, PARP12 can inhibit replication of RNA viruses (64). We found two presumed orthologs of human *PARP12* in ducks (LOC101802866 and LOC101796889, with the former being named *PARP12-L* for this paper). The shorter gene, *PARP12-L* is only significantly upregulated in lungs of VN1203 infected ducks, suggesting it may play a tissue specific role in viral inhibition. *PARP12* was also upregulated in a previous study in both lungs and ileums of VN1203 and BC500 infected Gray mallard ducks (25). Neither of these genes (*PARP12* or *PARP12-L*) has yet been characterized in birds, and it is unknown if either can restrict RNA viral replication. *PARP14* appears to be duplicated in the duck, with two forms of *PARP14* sharing equal percent identities to human *PARP14* (~43%), but only 52% identity to each other (data not shown). *PARP14* deletion reduces proinflammatory responses in murine macrophages (65), and in another study *PARP14* deletion was found to reduce IFN-β and ISG response (66). Both duck *PARP14* genes also have a predicted RNA binding domain, which is not present in the human *PARP14*. *PARP14.1* (LOC101789908) is upregulated by both BC500 and VN1203 infection in ducks, while *PARP14.2* (LOC101798744) is only upregulated by VN1203 in lungs and spleens of infected ducks. Members of the PARP gene family appear to be expanded in the duck, making them interesting candidates for further study of proteins which may play lineage specific roles in immune responses to IAV in the duck.

We postulate that specific responses to the virus that limit damage from infection may have been selected in ducks. Notably, lung tissues in VN1203 infected ducks show downregulation of some proinflammatory cytokines, including IL-17 and IL-8. In humans, IL-17 is elevated in patients who were infected with the 2009 S-OIV H1N1 IAV (67). Mice infected with the 2009 S-OIV H1N1 had a significant increase in survival when treated with anti-IL-17A monoclonal antibodies. In this study, we see a large increase of *IL17A* expression in spleens but not lungs of VN1203 infected ducks. Additionally, there was a significant decrease in expression of this gene, especially on 2 and 3 dpi, in the lungs of these infected ducks. This response may help lessen damage in the lungs from infection. We see a similar pattern in the proinflammatory cytokine IL-8, of which ducks have two presumed orthologous genes, *IL8* (LOC101804010) and *IL8-L* (LOC101803817). Both genes show strong upregulation in the spleen, particularly on 1 dpi. However, in lungs there is significant downregulation on 2 and 3 dpi of both *IL8* and *IL8-L*. In humans, IL8 is secreted by alveolar epithelial cells infected by IAV (68). As IL-8 is a potent neutrophil chemoattractant (68–70), decreasing the expression of the *IL8* gene in the site of VN1203 replication may reduce bystander damage to the tissues from excessive neutrophil accumulation. Interestingly, the downregulation of *IL17A*, *IL8* and *IL8-L* in lungs seems to be unique to our experiment. Huang et al. also investigated cytokine expression in lungs of Shaoxin ducks infected with DK/49, a HPAI H5N1 and GS/65 a LPAI H5N1 (23). In these experiments, *IL17A* was upregulated in the lungs of ducks infected with DK/49 on all three days, while it was downregulated in ducks infected with GS/65 on 1 dpi and increased in expression on 2 and 3 dpi (23). A similar pattern is seen in this data when comparing *IL8* and *IL8-L* expression. This is likely due to the viral strains used as well as the differences in breeds of duck. In our experiments all ducks infected with VN1203 survived. While Huang et al. do not specifically mention survival rates of the ducks used, Song et al. demonstrated that DK/49 killed all infected Shaoxin ducks with viral titres as low as 10^3^ EID_50_ (71). They also noted that GS/65 did not cause any mortality in infected ducks, yet it spread systemically in infected birds. Additionally, while Shaoxin ducks and Pekin ducks did originate from the same lineage, they have been selectively bred for eggs or meat (respectively) and inhabit separate clades in phylogenetic analyses (72, 73). It is also likely that this selective breeding has resulted in differences in immune responses.

As the intestine tissue both permits BC500 to reproduce to high titres, yet ultimately clears infection, we looked at both uniquely upregulated and downregulated gene expression, in comparison to other tissues. In intestines of BC500 ducks, we see upregulation of complement components *C1S* and *C4A* and strong downregulation of *C3* and *C5* as well as the complement receptor gene *CR1* and the *C5* receptor *CD88*. The C3 protein acts as a point of convergence to activate the classical, alternative and lectin pathways of complement activation [as reviewed by (74)]. Mice with *C3* and *CR1* genes knocked out were deficient in forming long term memory to IAV (75) while C5 activation is associated with lung damage during IAV infection in mice (76). Activated C5 is split into C5a, which is a potent chemoattractant of neutrophils (77) and monocytes (78). Limiting not only the key component of the complement cascade (C3) but also a potent activator of inflammatory cell subsets (C5) and their receptors likely decreases the inflammation in the intestine. Similarly, damage may be ameliorated by downregulation of *C3* and *C5* seen in lungs, but not spleens, of VN1203 infected ducks.

Because ducks are permissive to IAV replication while being resistant to pathology from replicating virus, we searched our data for genes that might assist in increased viral replication. In a recent review, Shaw and Stertz listed many genes that assisted in IAV replication in mammalian hosts (79), however many of these genes were not differentially expressed in ducks above physiologically relevant thresholds. Indeed, some of the differentially expressed genes that would allow for increased entry/endosome trafficking were downregulated in both intestines of BC500 ducks and lungs of VN1203 infected ducks. For example, *DYNLT2*, *ACTG2*, *ACTA1*, *ACTN2* and *ACTC1* were all downregulated in lungs of VN1203 ducks. Both actin and dynein proteins can aid in endosomal trafficking of IAV during early stages of infection (80). Several genes which encode chemoattractant proteins were specifically downregulated in intestines including Leukocyte cell-derived chemotaxin-2 (*LECT2*), a chemoattractant for neutrophils and macrophages (81, 82), *CCL26* a chemoattractant for eosinophils and basophils (83) and *IL15*, which has various functions in inflammatory responses and promoting immune cell maturation and proliferation (84). We did not take intestine samples from VN1203 infected ducks because the cloacal swabs of these ducks were negative. However, future research and analysis should include samples from tissues without virus present, to further elucidate which DEGs are from interferons and non-specific inflammatory responses, and which are caused by the presence of replicating virus.

Sex differences in immunity have evolved in all species from sea urchins to mammals, and where examined, innate and adaptive immunity is typically greater in females than males (85). Our study sampled a mix of male and female Pekin ducks in both our controls and infected birds. We observe some separation of samples according to sex in our MDS plot, thus it is possible that sex contributes to the differences in expression patterns seen, however this is mostly obscured by variation in response between genetically diverse individuals. Due to our relatively small sample size on each day of infection, we do not have enough male and female animals to compare immune responses by sex. Previously, we did not find differences in viral load between male and female ducks infected with rgVN1203 (26). In wild ducks, most studies show males carry more IAV (86–88), while one showed more female ducks infected (89), and one found differences in viral load between male and female ducks depending on geographic location (90). In the wild, host ecology contributes to prevalence of IAV infection in mallards including dabbling in infected water, flock density and migration (91).

Our results highlight the incredible complexity of tissue responses to both highly pathogenic and low pathogenic strains of IAV. Ducks are well equipped to control IAV replication, demonstrated by the shared expression of key IAV detectors and innate effectors in all tissues, notably the RIG-I pathway and interferon stimulated genes. The early timing of this robust early interferon response to VN1203 at 1 dpi may also be protective, while peak ISG responses are seen at 2 dpi for low pathogenic avian influenza. Many genes uniquely upregulated have as yet unknown roles in the physiological changes or immune response during infection, as thorough literature searches fail to link these genes to inflammatory modulators or viral restriction. It is suspected that recruitment of leukocytes contributes to DE of genes, but the responding cell types are not known. Ducks also have tissue-specific mechanisms in place to prevent damage and out-of-control inflammation, including downregulation of complement components *C3* and *C5*. The downregulation of certain proinflammatory genes, with the upregulation of other proinflammatory genes in the same tissues suggests the protection is from a dampening, rather than an all-out inhibition of the inflammatory response.

## Data Availability Statement

The datasets presented in this study can be found in online repositories. The names of the repository/repositories and accession number(s) can be found below: https://www.ncbi.nlm.nih.gov/sra/PRJNA767080.

## Ethics Statement

The animal study was reviewed and approved by the Animal Care and Use Committee of St. Jude Children’s Research Hospital and performed in compliance with relevant institutional policies, National Institutes of Health regulations, and the Animal Welfare Act.

## Author Contributions

LC performed the computational analysis and originally drafted and edited the manuscript. XF-C prepared RNA samples for sequencing. RW provided expertise and facilities for all infections and tissue collection. KM performed infections, collected tissues and RNA from infected ducks and edited the manuscript.

## Funding

Our research is funded by the Canadian Institutes of Health Research (PJT 159442) and the Natural Sciences and Engineering Research Council (to KM). All duck infections and isolation of infected tissues were supported by the National Institute of Allergy and Infectious Diseases, National Institute of Health under Contract No. HHSN27220140006C and by the American Lebanese-Syrian Associated Charities (ALSAC) to RW and conducted in Memphis Tennessee, USA.

## Conflict of Interest

The authors declare that the research was conducted in the absence of any commercial or financial relationships that could be construed as a potential conflict of interest.

## Publisher’s Note

All claims expressed in this article are solely those of the authors and do not necessarily represent those of their affiliated organizations, or those of the publisher, the editors and the reviewers. Any product that may be evaluated in this article, or claim that may be made by its manufacturer, is not guaranteed or endorsed by the publisher.

## Supplementary Material

Supplementary Figure 1 Gene ontology (GO) analysis of alternatively spliced genes in VN1203 infected ducks. Statistically significant (FDR < 0.05) alternatively spliced genes were submitted to enrichment analysis and the top 10 most enriched terms in the category of GO biological process are graphed for lungs of ducks infected with VN1203 on 1 dpi **(A)** and lungs and spleens of ducks infected with VN1203 on 3 dpi **(B)**.

Supplementary Figure 2 Top 100 downregulated genes in lungs of VN1203 and intestines of BC500 infected ducks. Lists of statistically significant (FDR<0.05) genes were filtered for the 100 genes most downregulated in lungs of VN1203 infected ducks on 1 dpi, and intestines of BC500 ducks on 2 dpi. Redundant genes, pseudo genes and genes denoted as ncRNA in NCBI were manually removed from lists.
