# Supplementary figures and images for: Tissue Specific Transcriptome Changes Upon Influenza A Virus Replication in the Duck
Source: Front Immunol. 2021 Nov 5;12:786205. doi: 10.3389/fimmu.2021.786205 (PMC8602823; doi:10.3389/fimmu.2021.786205)

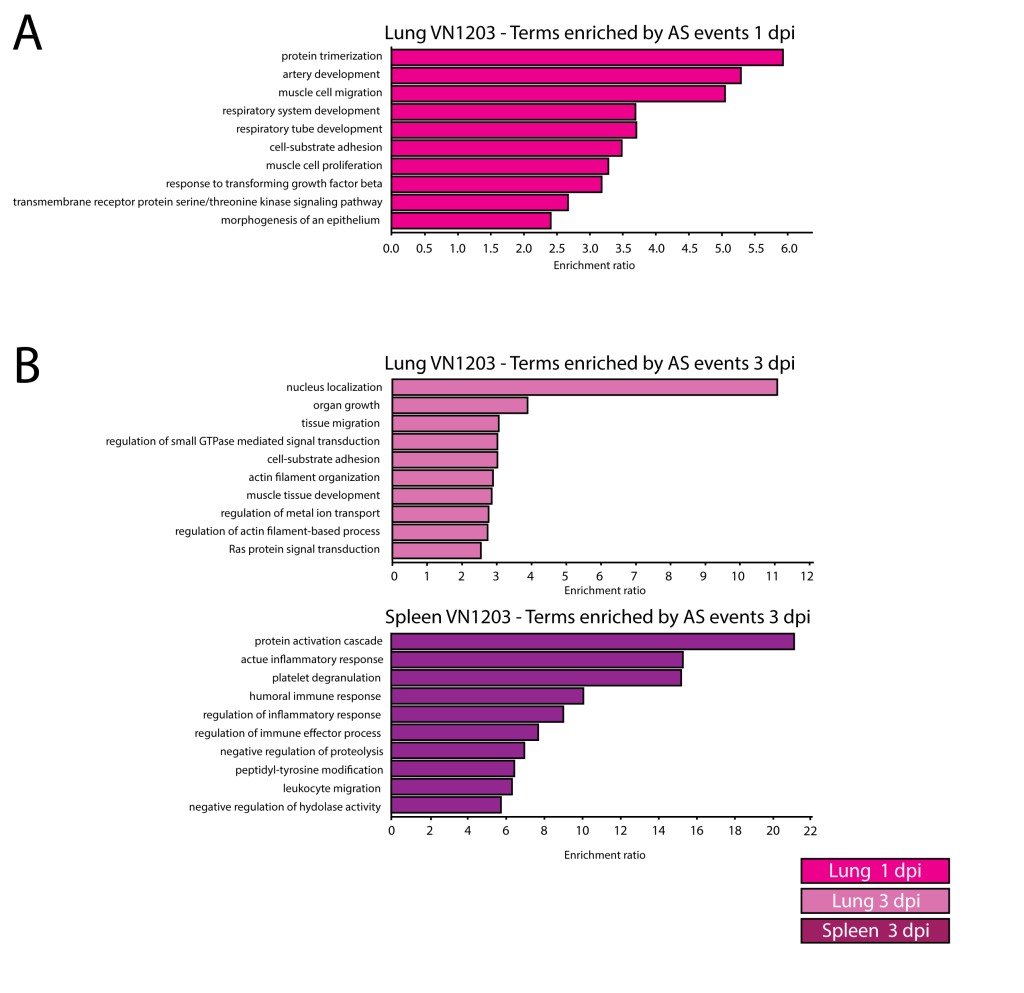

Supplement: Supplementary Figure 1 — Gene ontology (GO) analysis of alternatively spliced genes in VN1203 infected ducks. Statistically significant (FDR < 0.05) alternatively spliced genes were submitted to enrichment analysis and the top 10 most enriched terms in the category of GO biological process are graphed for lungs of ducks infected with VN1203 on 1 dpi (A) and lungs and spleens of ducks infected with VN1203 on 3 dpi (B). [file Image_1.tif]

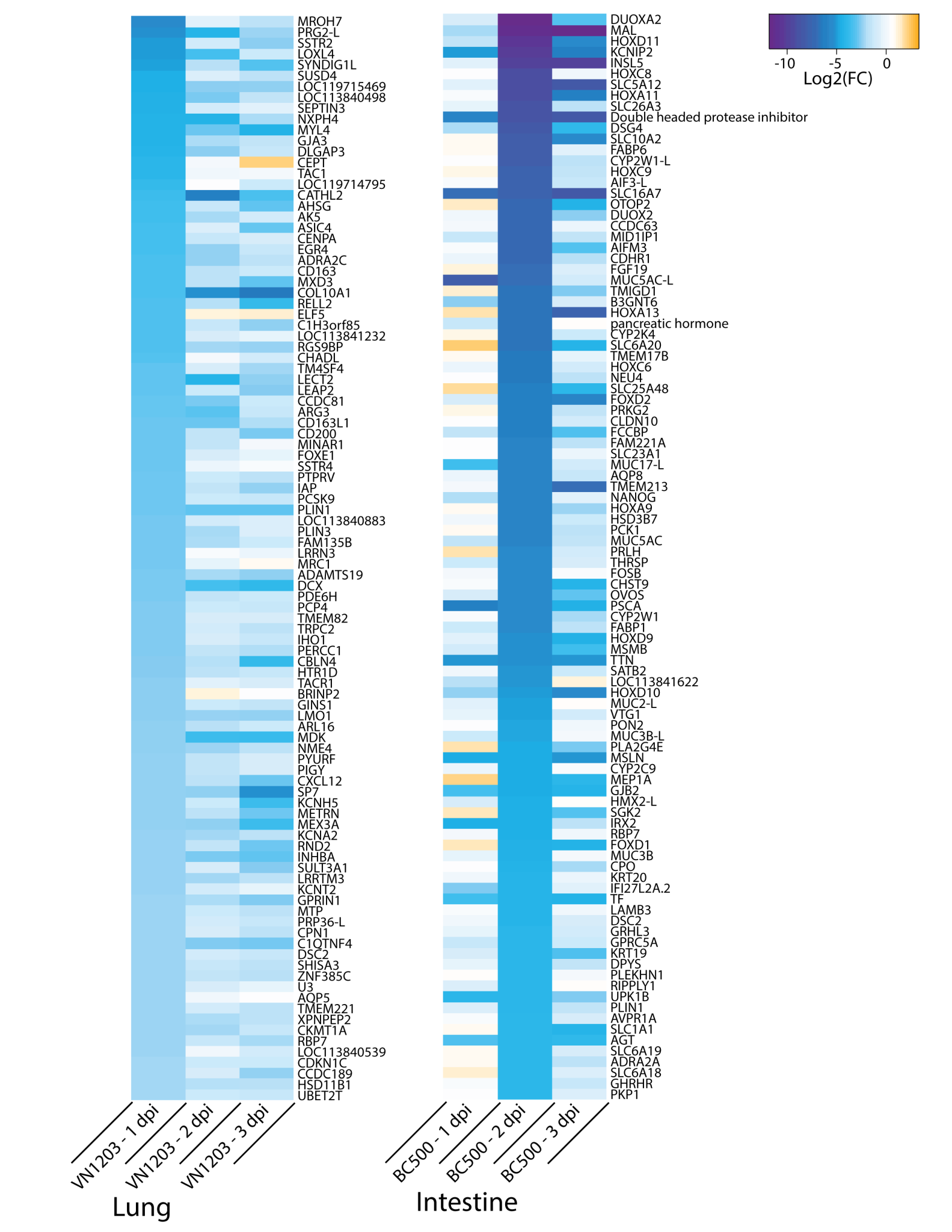

Supplement: Supplementary Figure 2 — Top 100 downregulated genes in lungs of VN1203 and intestines of BC500 infected ducks. Lists of statistically significant (FDR<0.05) genes were filtered for the 100 genes most downregulated in lungs of VN1203 infected ducks on 1 dpi, and intestines of BC500 ducks on 2 dpi. Redundant genes, pseudo genes and genes denoted as ncRNA in NCBI were manually removed from lists. [file Image_2.tif]
